# Adults interpret iconicity in speech and gesture via the same modality-independent process

**DOI:** 10.3758/s13423-025-02698-2

**Published:** 2025-05-12

**Authors:** Mingtong Li, Suzanne Aussems, Sotaro Kita

**Affiliations:** Department of Psychology, University of Warwick, Coventry, CV4 7AL UK

**Keywords:** Iconicity, Prosody, Co-speech gestures, Speed

## Abstract

Iconicity is the resemblance or similarity between the form of a signal and its meaning. In two studies, we investigated whether adults interpret iconicity in speech and gesture via a modality-independent mechanism (Study 1, *N* = 40; Study 2, *N* = 348). Participants in both studies completed two verb–action matching tasks. In these tasks, either spoken verbs or co-speech gestures were manipulated at different rates to iconically represent actions (i.e., manners of locomotion) performed at corresponding rates. We hypothesized that adult participants would map verbs spoken at a fast rate to fast actions and verbs spoken at a slow rate to slow actions. Similarly, we hypothesized that adult participants would map gestures produced with faster hand movements to fast actions and gestures produced with slower hand movements to slow actions. Moreover, we predicted a positive correlation between the two task performances. In both a pilot study (Study 1) and a preregistered replication (Study 2), participants successfully used iconic cues to match verbs to actions in both verb–action matching tasks. Crucially, adult participants who performed well in the verb–action matching task with iconic speech cues also performed well in the verb–action matching task with iconic gesture cues. This weak positive correlation remained significant even after controlling verbal working memory performance. These findings suggest that adults may rely on a modality-independent cognitive mechanism for interpreting iconicity both within and across different sensory modalities, using both visual information from gestures and auditory information from speech to identify referent actions.

**Supplementary Information:**

The online version contains supplementary material available at 10.3758/s13423-025-02698-2.

Iconicity is the resemblance between the form of a signal and its meaning, where the form shares perceptual or structural similarities with the meaning it represents (Dingemanse et al., 2015; Emmorey, 2014; Taub, 2001). Iconic mappings span sensory modalities, such as auditory and visual, and are expressed in various communicative forms, including verbal forms like spoken words and nonverbal forms like co-speech gestures (e.g., Perniss & Vigliocco, 2014). For instance, in the auditory modality, sound-symbolic words can convey iconic information for physical size, where high front vowels (e.g., /i/) are generally assumed to represent smaller objects, and low back vowels (e.g., /ɑ/) to larger objects (Sapir, 1929; Winter et al., 2022), possibly because the oral cavity is narrower for high front vowels than low back vowels. Similarly, in visual modality, larger hand movements iconically represent larger objects, while smaller hand movements represent smaller ones (Beattie & Shovelton, 2002; Holler et al., 2009). Thus, different sensory modalities can iconically convey the same referent features (e.g., physical object size), suggesting that iconicity transcends sensory modalities. Given that iconicity can convey the same referent features across different sensory modalities, our research question is whether the interpretation of iconic mappings involves a modality-independent mechanism.

Interpreting iconicity involves identifying perceptual features from sensory input and aligning those with mental representations of the referent (Emmorey, 2014). These mappings can occur either within a single sensory modality or across multiple modalities. Unimodal mappings involve processing signals and referents within the same sensory modality. For example, when people hear the word “*buzzing*”, this might evoke the sound of a bee via iconicity (Winter et al., 2022). Similarly, when interpreting iconic gestures, such as hand gestures depicting the shape or motion of an object (e.g., fingers moving alternately to mimic walking legs), adults must align the visual features of the hand gesture with the visual features of the corresponding action (Beattie & Shovelton, 2002; Dargue et al., 2019) within the visual modality.

Iconic mappings can also occur cross-modally. Cross-modal mappings involve processing signals and referents across different sensory modalities. For example, in sound-symbolism research, the “*maluma–takete*” effect (Köhler, 1947) demonstrates how people associate words with /u/ and liquid and nasal consonants (e.g., “*maluma*”) with rounded shapes and words with /e/ and voiceless stop consonants (e.g., “*takete*”) with spiky shapes (also known as the “*bouba–kiki*” effect; Ramachandran & Hubbard, 2001). This effect involves both the auditory (spoken words) and visual modalities (shape images). Similarly, iconic gestures can also facilitate cross-modal mappings. For example, in musical performances, a conductor’s widening arm gesture is used to visually signal a gradual increase in volume (Poggi & Ansani, 2016). In this gesture, the conductor starts with their arms closer together and then slowly extends them outward while raising the shoulders upward. The gesture visually represents the expansion or “widening” of the sound, in other words, a larger volume.

We propose that a modality-independent cognitive mechanism allows adults to interpret iconic mappings, both within and across different sensory modalities. That is, when interpreting iconic cues, adults first have modality-specific cognitive processes to extract and organize information—such as the acoustic features of speech or music, or the visual features of gestures, movements, or shapes—into schematic representations. A subsequent modality-independent process then compares these representations across modalities, allowing adults to link iconic cues and their referents based on perceived similarity or structural resemblance (e.g., “maluma” sounds like a round object).

This proposal is further motivated by the finding that the “bouba-kiki” effect extends beyond the shape domain, encompassing also other types of information such as emotion (Kantartzis, 2011). People judged words with stop consonants to match referents that involve abrupt changes, such as spikey shapes (in which the outline can be seen as changing the direction of its tangent abruptly), abrupt change of color, and abrupt change of emotion. Conversely, they judged words with liquids and nasals to match referents that involve gradual changes, such as round shapes, gradual change of color, and gradual change of emotion. This suggests that the acoustic and articulatory information of words can be converted into a modality-independent representation of temporal patterns of change. For example, stop consonants involve more abrupt articulatory and acoustic changes compared with liquids and nasals (see also Imai & Akita, 2023). This abstract representation of temporal patterns can then be mapped onto a range of referents, not just shapes, but also those involving various abrupt versus gradual changes in color, emotion, and other domains.

## The current research

We investigated whether adults interpret iconicity in speech and gesture through a modality-independent mechanism. Specifically, we tested their ability to map novel spoken verbs to visual actions using iconic prosody (cross-modal mapping) and iconic gesture (unimodal mapping). We hypothesized that adults who perform well on cross-modal mappings (e.g., auditory-to-visual) would also excel at unimodal mappings (e.g., visual-to-visual), and vice versa. A positive correlation between these performances would provide evidence for a modality-independent cognitive mechanism for processing iconicity.

We first conducted an in-person pilot study with a smaller sample (Study 1, *N* = 40) and then preregistered and replicated the results in an online study with a larger sample (Study 2, *N* = 348). Both studies examined whether adults interpreted iconicity similarly in speech (cross-modal mappings) and gesture (unimodal mappings). To test this, we designed two verb–action matching tasks where participants were presented with either iconic speech or iconic gesture cues. In each task, participants watched a pair of videos showing either a boy or a girl performing computer-modified fast and slow versions of the same action. A third video introduced a novel verb, with an actress engaging participants and providing iconic cues while saying, “Look! The boy/girl is *daxing*!”. Participants were then required to select the action video they believed best matched the verb. We measured how often participants selected the target action that matched the speed of the iconic cue (fast or slow rate).

## Predictions

In the verb–action matching task with iconic speech cues, adult participants heard iconic prosodic cues embedded in the novel verbs without seeing iconic gesture cues. We expected participants to match fast speech rates to the fast action version and slow speech rates to the slow action version. If this iconic prosody effect is present, their average proportion of target action choices should be above chance (Studies 1–2, H1a).

In the verb–action matching task with iconic gesture cues, adult participants saw iconic gestures that accompanied the novel verbs without hearing iconic speech cues. We expected participants to match fast gesture rates to the fast action version and slow gesture rates to the slow action version. If this iconic gesture effect is present, their average proportion of target action choices should be above chance (Studies 1–2, H1b).

Crucially, our main prediction was that adult participants who perform well in the verb–action matching task with iconic speech cues would also perform well in the verb–action matching task with iconic gesture cues (Studies 1–2, H2a), even after controlling verbal working memory (Study 2, H2b).

## Study 1

### Method

Study 1 was a pilot study and was therefore not preregistered. However, it served as a foundation for a preregistered replication in Study 2. The raw data and materials are available via the Open Science Framework (OSF; https://osf.io/4mnqg/).

#### Design

This study employed a mixed-design approach, combining an experimental manipulation and a correlational design to investigate a modality-independent mechanism for interpreting iconicity. The experimental manipulation involved varying the speed of iconic cues in two verb–action matching tasks, one for speech and one for gesture. In the verb–action matching task with iconic speech cues, spoken verbs were computer-modified to be fast, normal, or slow. In the verb–action matching task with iconic gestures, the speed of hand movements was manipulated in the same way. Each task included four trials with fast cues, four trials with slow cues, and four filler trials with normal-speed cues (12 trials in total). Normal-speed cues reflected the natural rate of a native English speaker and were not designed to iconically represent fast or slow actions.

In each trial, participants completed a two-alternative forced choice task, selecting the action video (fast or slow) they believed best matched the verb. Choices were coded as target if the selected action rate matched the iconic cue, and as distractor if it did not. Target action choices for the fast and slow conditions were averaged across trials to compute a proportion of target choices for each task. Normal-speed trials were excluded from this calculation, as they were not part of the experimental manipulation.

Although the two verb–action matching tasks involved an experimental manipulation of speed in the fast and slow conditions, the main hypothesis was correlational. Specifically, this study examined the relationship between two key variables: (1) the proportion of target action choices in the verb–action matching task with iconic speech cues; (2) the proportion of target action choices in the verb–action matching task with iconic gesture cues.

#### Participants

The final sample included 40 adult participants (8 males, 32 females) through convenience sampling from the first-year undergraduate participant pool at the University of Warwick. Adult participants were on average 18.70 years old (*SD* = 0.65), ranging between 18 and 20 years. The final sample’s ethnic composition was as follows: 28 participants identified as White, 3 as Black (or Black British, Caribbean, or African), 7 as Asian (or Asian British), 1 as Mixed (or from Multiple Ethnic Groups), and 1 chose not to disclose their ethnicity. All participants were fluent in English, either as native speakers or as individuals who had studied in an English-speaking country for at least 2 consecutive years. The average self-reported exposure to English of all time was 92.42% (*SD* = 10.86%). Participants received course credits as compensation for their time. The study protocol was approved by the Department of Psychology Ethics Committee at the University of Warwick (reference: PSY_PGR_22-23/28).

An additional nine participants were tested but excluded from the analysis for the following reasons: (1) they were non-native English speakers who reported less than 2 consecutive years of having studied in an English-speaking country (*n* = 7), or (2) they experienced technical issues due to the experimental program crashing (*n* = 2).

#### Materials

##### Speech stimuli

Speech stimuli were recorded on a MacBook Pro 2020 by a female native English speaker, who was blind to the study design and hypotheses. The voice actress recorded the carrier sentence, “Wow! Look at what is happening! The boy (or the girl) is *novel-verbing*. Which one is *novel-verbing*?”, with 12 different novel verbs embedded into the carrier sentence. The novel verbs she recorded were *daxing, blicking, glabbing, howning, krading, larping, mipping, pilking, poffing, stumming, wepping*, and *yoofing*. These novel verbs follow the rules of the English language and are widely used in the verb learning literature (Aussems & Kita, 2021; Aussems et al., 2022; Childers, 2011; Mumford & Kita, 2014; Mumford et al., 2023).

The voice actress was instructed to vary the speech rate of the novel verb, saying it at a fast, normal, and slow rate, while keeping the carrier sentence at a consistent (normal) speech rate across all sentences. She recorded each carrier sentence incorporating a novel verb consecutively five times. We did not prescribe any particular speech rate for the carrier sentence in the three conditions; the native speaker spontaneously determined the speech rate in the three conditions. This resulted in 30 sentences per novel verb, with 15 sentences describing a male actor (“The boy is *novel-verbing*”) and 15 sentences describing a female actor (“The girl is *novel-verbing*”) in the action video. Each set of 15 sentences consisted of five sentences at fast, normal, and slow speech rates. Then the novel verbs in all takes were manually segmented out of the carrier sentence using Praat speech analysis software (Version 6.1.16; Boersma & Weenink, 2024) and saved as separate audio clips.

Our goal was to create ecologically valid speech stimuli when manipulating the speech rate of the novel verbs. To achieve this, we followed a systematic approach to determine the speech rate for the novel verb in each condition. First, we calculated the average time durations of the novel verb clips in the five recorded sentences, grouped by speed conditions and the gender of the actor in the action video clips. Next, we identified a verb clip from the normal speed condition that had the smallest deviation from the average time duration in that same condition. This clip served as the baseline clip, and the carrier sentence recorded alongside it was chosen as the carrier sentence for the novel verb across all three speed conditions. This ensured that only the speed of the verb varied, while the carrier sentence remained identical for a given verb and a given gender of the referent actor (“... The boy is *novel-verbing*...” or “... The girl is *novel-verbing*...”). Second, to modify the baseline clip and create the desired verb clip to be inserted into the carrier sentence, we used Adobe Audition 2022. Specifically, we used the “Stretch and Pitch (process) Effects” feature to adjust the stretch percentage based on the average time durations for the verb in the fast and slow conditions. The pitch shift was set to zero semitones. This approach preserved linguistic features such as intensity and pitch, ensuring that only the speech rate of the verb clip was manipulated across conditions. Importantly, the speech rate modifications applied to the verb clips were based on the natural average acceleration and deceleration rates specific to each novel verb, making the speech stimuli sound as natural as can be. Finally, the modified verb clips were inserted into the baseline clip’s carrier sentences, and each sentence was concatenated with a 1.5-s pause in between. This ensured that the same verb shared the same carrier sentence across the conditions, while modifying only the speech rate of the novel verb. For the average time durations and the acceleration and deceleration rates of each novel verb, please refer to the OSF documentation.

##### Action video stimuli

A set of 12 action video clips was selected from the GestuRe and Action Exemplar (GRACE) video database (Aussems et al., 2017, 2018). In the action video clips, an actor performed an unusual manner of locomotion to get from one location to another (i.e., from the left side to the right side of a scene), which looked like a funny manner of walking or jumping. The speed of selected locomotion manners was modulated using Adobe Premier Pro, to create a fast version and a slow version of each action video. The acceleration and deceleration rates were the same as those of the novel spoken verbs. This modification ensured that the spoken novel verbs and action videos shared the same acceleration or deceleration rates, as we expect systematic speed modifications to facilitate iconic mappings between the speech rate of novel verbs and the referent actions.

##### Gesture video stimuli

**Fig. 1:**
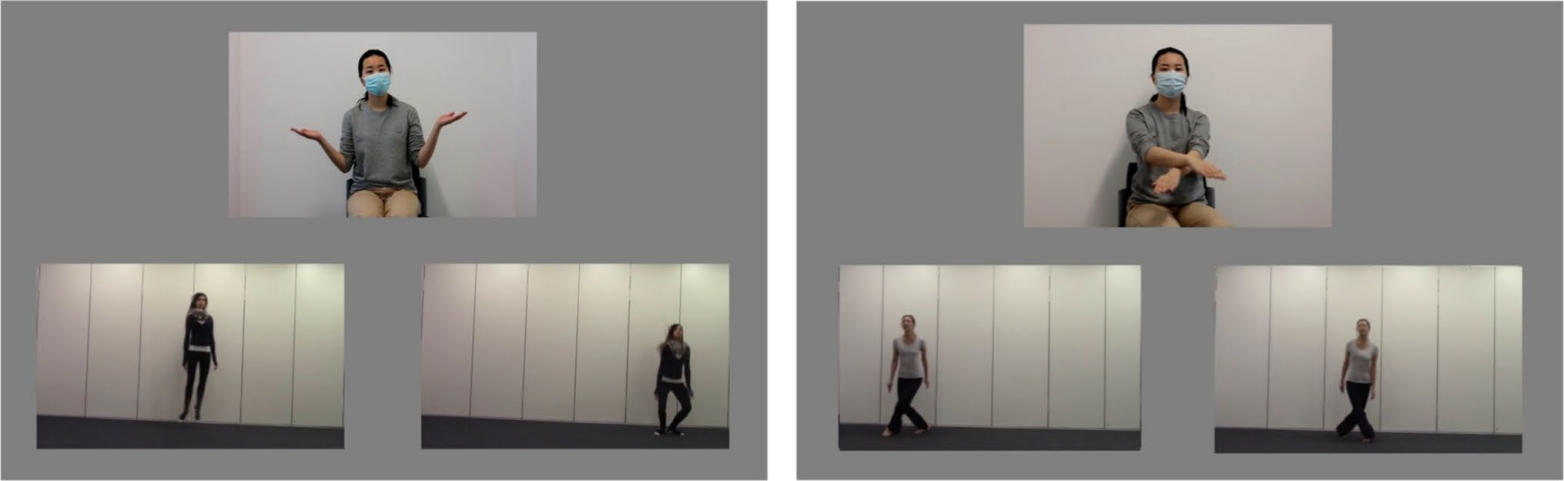
Example of the test trials in the two verb–action matching tasks. *Note.* The figure shows two examples of the three videos that a participant saw simultaneously on the screen in a test trial in verb–action matching tasks with iconic speech cues (left) and with iconic gesture cues (right). In the iconic speech cue task, the actress produced interactive gestures (e.g., a palm-up open-hand gesture). In the iconic gesture cue task, the actress produced iconic gestures depicting the manner of actions (e.g. crossing her arms to depict the leg-crossing movements in the action videos). The speed of the iconic gesture was matched to one of the two action speeds (fast or slow)

A set of 12 iconic gesture templates for the selected action video clips were chosen from the same GRACE video database to be used in the iconic gesture cue task. The iconic mappings between action videos and corresponding iconic gestures have been normed and validated with adult participants (Aussems et al., 2017, 2018), as well as used in experiments with preschool-aged children (Aussems et al., 2022; Aussems & Kita, 2021). Gesture video clips were recorded again specifically for this study using a Canon R6 camera, following those validated iconic gesture templates. To ensure consistency across the different speech stimuli, the actress in our video wore a surgical face mask during recording. This concealed her mouth movements, preventing any mismatch between her lip movements in the videos and the audio of the speech stimuli presented to participants. In the gesture video clips, the actress performed an iconic gesture, depicting an action repeatedly (see Fig. 1, the right panel). The gesture rate aligned with the moving rate of the manner of locomotion in the action video. The speed of the gesture videos was then computer modified in the same way as the speed of the action videos, to create a fast and a slow version of each gesture video. In the iconic speech cue task, we also showed videos of an actress producing interactive gestures (e.g., a palm-up open-hand gesture often produced with a question). The interactive gestures did not encode any information about the target action (the left panel of Fig. 1).

##### Stimulus pairings

A total of 12 pairings were made, each consisting of an action and a novel verb. The same action was always paired with the same novel verb. For a full list of the stimulus pairings, please refer to the OSF documentation.

##### Software and apparatus

The verb–action matching tasks were programmed in PsychoPy (Peirce et al., 2019) carried out on a MacBook Pro 2020.

#### Procedure

The study was conducted in person in a psychology research lab at the University of Warwick. All participants completed the same two tasks in the same order. First, participants completed a verb–action matching task with iconic gesture cues (and no iconic speech cues). During this task, participants completed 12 two-alternative forced-choice trials: four filler trials with normal speed cues and 8 experimental trials with fast or slow cues. In each trial, participants were presented with two video clips of the same actor who moved across the scene in a funny and unusual manner. One video showed a computer-modified slow version of the action, and the other video showed a computer-modified fast version of the same action. Simultaneously, participants were also presented with a third video clip, featuring an actress who sat on a chair wearing a face mask, accompanied by recorded speech introducing a new verb, “Wow! Look at what is happening! The boy (or the girl) is *novel-verbing*!”. Simultaneously, the same actress also produced a hand gesture at a fast, normal, or slow rate. The fast and the slow rate gestures could be mapped iconically onto the fast or slow action videos. The normal rate gestures were not manipulated to match either video and therefore served as filler trials, offering no clear match to either. After the actress said the novel verb, participants were asked to select one the action video that best matched the novel verb, “Which one is *novel-verbing*?”.

Next, participants completed a verb–action matching task with iconic speech cues (without iconic gesture cues). This task followed the same structure as the verb–action matching task with iconic gesture cues. Instead of gestures, the actress provided the iconic cue about the speed of the referent action through speech rate. The fast and slow speech rates iconically mapped onto the fast and slow action videos. The forced-choice task procedure was the same as in the verb–action matching task with iconic gesture cues. An example of what participants saw during the experiment is presented in Fig. 1.

#### Counterbalancing and randomization

The position of the target action was counterbalanced across trials. Half the time the target action appeared on the left side of the screen and half the time on the right side of the screen. The speed of the target actions was also counterbalanced across trials. One-third of the time the target actions were fast, one-third were normal, and one-third were slow. Furthermore, whether the fast or slow version of an action was the target action was counterbalanced across two versions of the task. For a specific action, one version of the task included the fast version as the target action and the other version included the slow version. The order of trials was randomized for each participant using PsychoPy (Version 2023.1.2).

#### Data analysis

We first created two key variables, the proportion of target action choices, for both verb–action matching tasks respectively (with iconic speech or iconic gesture cues). For each trial in the fast and slow speed conditions, participants received a score of 1 for selecting the target action, and a score of 0 for selecting the distractor action. These scores were then summed across all trials and divided by the total number of test trials in the fast and slow conditions (*n* = 8) to create a proportion of target action choices per verb–action matching task. For instance, if a participant chose the target action in six out of eight trials in the fast and the slow speed conditions, the proportion of the target action choices would be .75.

To test H1a and H1b, we conducted two separate one-sample *t* tests (two-tailed) to examine the iconic effects in both verb–action matching tasks. These tests compared the average proportion of target action choices in each task against chance (test value = .50).

To test H2a, we conducted a Spearman’s rank correlation analysis to examine the relationship between the average proportions of target action choices in the two verb–action matching tasks. Spearman’s rank correlation was chosen due to the nonnormal distribution of the proportion data. Bootstrapping was applied to obtain robust 95% confidence intervals around the correlation coefficient, particularly given the relatively small sample size in this study.

All analyses were conducted with R (Version 4.2.2) and R Studio software (Version 2023.06.2) for statistical analysis. The following R packages were used: *dplyr* (Wickham et al., 2023) and *stringr* (Wickham, 2022) for data wrangling, *ggplot2* (Wickham, 2016) and *ggpubr* (Kassambara, 2020) for data visualization, *rstatix* (Kassambara, 2021) for one-sample *t* tests, and *rcompanion* (Mangiafico, 2023) for Spearman’s rank correlation and 95% bootstrapped confidential intervals.

### Results

#### Chance comparisons

**Fig. 2:**
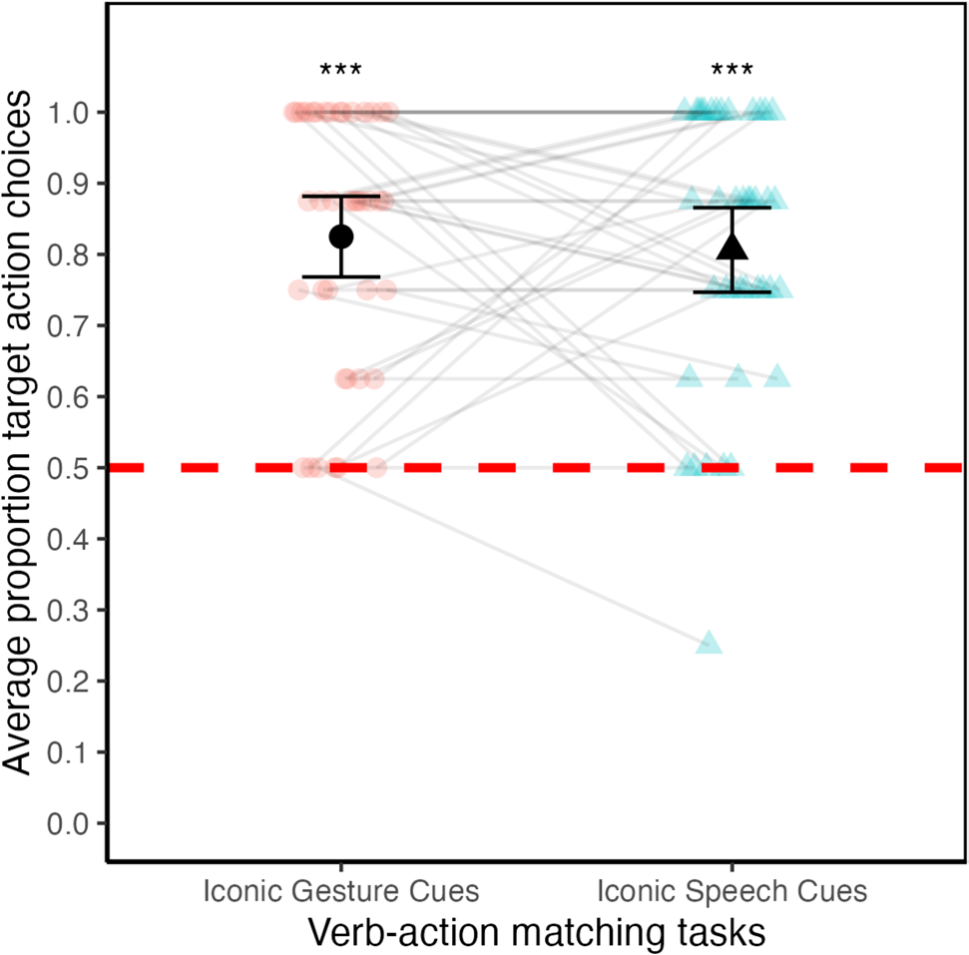
Average proportion target action choices by verb–action matching tasks (Study 1, *N* = 40). *Note.* Average proportion target action choices (*y*-axis) by verb–action matching tasks (*x*-axis). The black shapes (circle, triangle) represent the means of all adult participants. Faded shapes represent individual performances, with light gray lines connecting the performances of the same individuals across the two verb–action matching tasks. Error bars represent the 95% confidence intervals around the means. The dashed red horizontal line represents the chance level. Asterisks (***) represent the significance (*p* < .001) for each comparison of the mean performance in each verb–action matching task with chance. *N* = 40 adult participants (Study 1). (Color figure online)

To test H1a and H1b, we first compared adults’ average proportion of target action choices against chance (test value = 0.50) for each verb–action matching task. Figure 2 shows adults’ target action choices (in proportion) organized by verb–action matching task. Adults’ average proportion of target action choices of .83 (*SD* = .18) in the verb–action matching task with iconic gesture cues was significantly above chance, *t*(39) = 11.23, *p* < .001, 95% CI around the mean [.77, .88]. The magnitude of this effect was large, Cohen’s *d* = 1.78, 95% CI *d* [1.35, 2.56]. Adults’ average proportion of target action choices of .81 (*SD* = .19) in the verb–action matching task with iconic speech cues was also significantly above chance, *t*(39) = 10.09, *p* < .001, 95% CI around the mean [.74, .87]*.* The magnitude of this effect was large, Cohen’s* d* = 1.60, 95% CI *d* [1.09, 2.40]. These results were based only on the fast and slow speed conditions, as the normal speed condition did not indicate a particular target action. Analyses comparing performance against the normal speed trials, as well as comparisons between the iconic speech and iconic gesture tasks are included in the Supplementary Materials.

#### Correlation

**Fig. 3:**
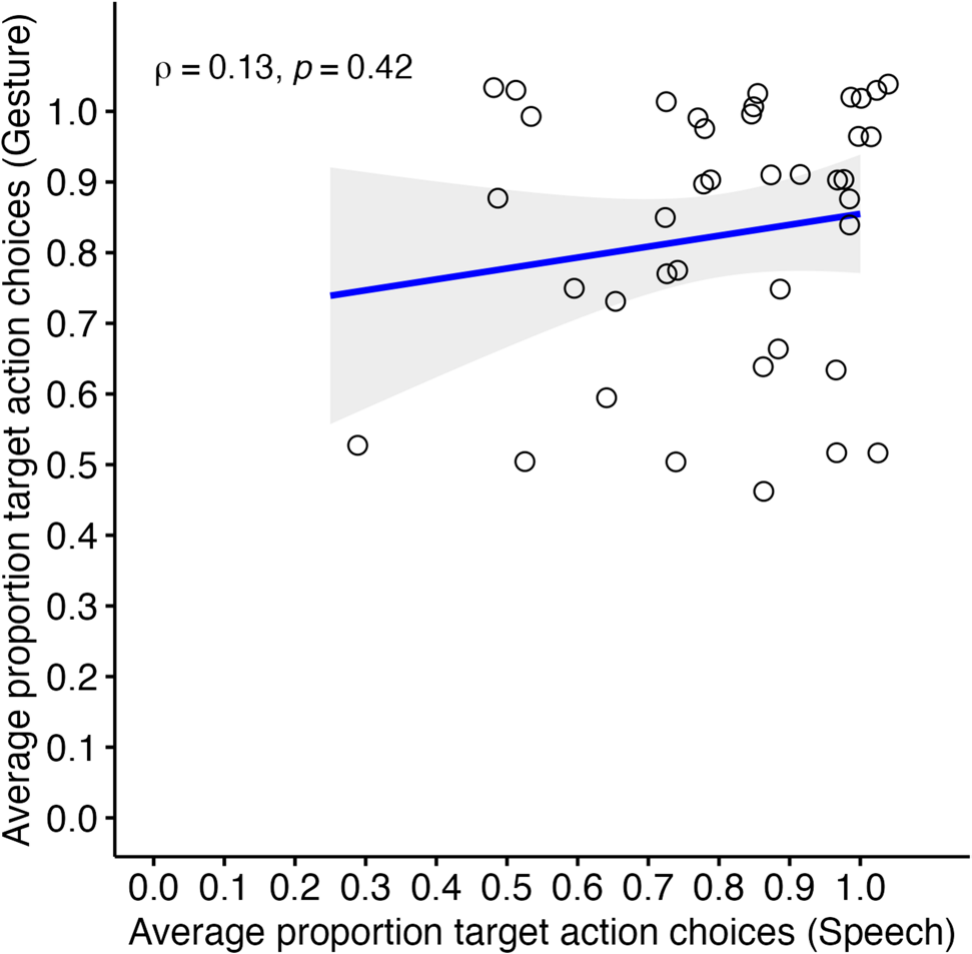
Spearman rank correlation of adults’ performances in the two verb–action matching tasks (Study 1, *N* = 40). *Note.* Scatterplot of the average proportion target action choices in the verb–action matching tasks with iconic gesture cues (*y*-axis) and iconic speech cues (*x*-axis). The jittered blank dots represent individual performances in both tasks. The thick blue line indicates the line of best fit, with the gray shaded area representing the 95% confidence intervals. *N* = 40 adult participants (Study 1). (Color figure online)

To test H2a, we correlated adults’ performances between the two verb–action matching tasks. Figure 3 illustrates the relationship between the average proportions of target action choices in the verb–action matching tasks with iconic speech cues and iconic gesture cues. There was a weak positive but nonsignificant correlation between verb–action matching tasks with iconic speech cues and iconic gesture cues, *ρ*(38) = .133, *p* = .415, bootstrapped 95% CI *ρ* [− .195, .443].

### Discussion

Study 1 provided two important preliminary insights. First, the adult participants were indeed able to select target actions above chance in both verb–action matching tasks, showing large and significant effects for both iconic speech and iconic gestures, confirming H1a and H1b. Adults have a good understanding of iconic speech and gesture cues, validating our stimulus sets and tasks.

Second, the weak positive but nonsignificant correlation indicated a trend toward a subtle modality-independent ability, though there was insufficient evidence to confirm H2a. The small effect size and lack of significance suggested that the sample size might not have provided enough statistical power to detect such an effect. Additionally, several methodological issues may have contributed to the weak positive correlation. One possibility is that using the same verb–action pairings across both tasks introduced memory interference, with participants’ choices in the second task possibly being influenced by their responses in the first task. Furthermore, the correlation between the two tasks could partly reflect individual differences in general cognitive ability, which may have introduced noise and not specifically measured the ability to interpret iconicity.

## Study 2

Study 2 aimed to address the limitations of Study 1 by increasing the sample size and refining the method. To better isolate the effect specific to iconicity interpretation, we added a verbal working memory task as a control task, which was unrelated to the verb–action matching tasks (Nicoladis & Gagnon, 2020; Wu & Coulson, 2015), which we presumed to have no iconicity interpretation components. This control task allowed us to determine whether the positive relationship observed between the two verb–action matching tasks was specific to iconicity interpretation, rather than influenced by verbal working memory or other irrelevant factors (e.g., general intelligence and levels of engagement with an online experiment). We chose not to include a visual working memory task because iconic gesture comprehension positively correlates with visual working memory, but not with verbal working memory (Wu & Coulson, 2014a, 2014b). Such results suggest that visual working memory tasks might have iconicity interpretation components like similarity-based mappings; thus, a verbal working memory task was a better choice as the control task. Additionally, we refined the methodology in three ways: 1) verb–action pairings were varied between tasks to minimize memory interference, 2) task order was reversed to ensure that results do not depend on the sequence of tasks, and 3) normal-speed filler trials were removed to focus exclusively on the fast and slow speed trials, which directly tested our iconicity effects with a more efficient design.

### Method

The hypotheses, methods, materials, and analyses were preregistered via OSF prior to data collection (https://osf.io/jp6qn/). The raw data and analysis scripts are available via OSF (https://osf.io/4mnqg/).

#### Design

The design of Study 2 was the same as in Study 1, except for the following. We added a forward digit span task between the two verb–action matching tasks to control participants’ verbal working memory (adapted from Roembke & McMurray, 2021). In this task, participants were asked to remember and recall 4 sequences of 8 digits in order of appearance. Their performance was scored for each sequence, and an average score was calculated across all sequences.

#### Power analysis

A power analysis was conducted using G*Power (Faul et al., 2009) to estimate the sample size needed to detect a weak but significant positive correlation. Based on the findings of Study 1 (pilot): a correlation coefficient of 0.133, a significance level (*α*) of 0.05, and a power of 80%, the required sample size was estimated to be 348.

#### Software and online platform

The study was conducted online via the crowdsourcing platform Prolific Academic. The verb–action matching tasks were programmed in PsychoPy (Peirce et al., 2019) and run using Pavlovia (Bridges et al., 2020). To ensure high-quality data, participants were prescreened via Prolific Academic. Prescreening criteria included 1) English as the first language and primary language, 2) no language-related disorders, 3) no literacy difficulties, and 4) a previous study payment approval rate of 99% or higher. Prescreening settings also included a representative gender-balanced sample.

#### Participants

The final sample included 348 adult participants (169 males, 177 females, and 2 non-binaries) recruited via Prolific Academic. Adult participants were on average 43.84 years old (*SD* = 13.40), ranging between 18 and 76 years. The final sample’s ethnic composition was as follows: 296 participants identified as White, 22 participants as Black (or Black British, Caribbean or African), 21 participants as Asian (or Asian British), 7 participants as Mixed (or Multiple Ethnic Groups), and 2 participants did not disclose their ethnicity. All participants were located in the United Kingdom and reported English as their first and primary language. Participants were paid a rate of £9 per hour for their time. The study was approved by the Department of Psychology Ethics Committee at the University of Warwick (reference: PSY_PGR_22-23/28).

An additional 41 participants were tested but excluded from the analysis for the following reasons: (1) They showed a speed bias and selected exclusively on answers of the same speed in the verb-matching tasks (*n* = 19); (2) they failed at least two out of three attention checks (*n* = 2); (3) they reported writing down digits or using external aids to complete the forward digit span task (*n* = 6); (4) they did not report English as their first and primary language (*n* = 12); or (5) they failed several of the abovementioned checks (*n* = 2). Excluded participants were replaced by new participants to achieve sufficient statistical power with the estimated sample size of 348 participants.

#### Materials

The materials used in Study 2 were the same as in Study 1, except for two modifications. Based on the results of Study 1, we assigned six of the 12 action–verb pairings exclusively to the verb–action matching task with iconic speech cues, and the remaining six pairings to the verb–action matching task with iconic gesture cues. This ensured that no verb–action pairings appeared in both tasks, minimizing potential memory interference. Each verb–action pairing was repeated twice within its respective task, resulting in a total of 12 trials, consistent with Study 1. Second, we dropped the normal-speed filler trials.

#### Procedure

The procedure was similar to Study 1, but the order of the verb–action matching tasks was reversed. All participants completed the same three tasks in a fixed order: a verb–action matching task with iconic speech cues, a forward digit span task (assessing verbal working memory), and a verb–action matching task with iconic gesture cues.

First, participants completed a verb–action matching task with iconic speech cues (without iconic gesture cues). This task included six unique verb–action pairs, with each verb–action pair appearing in both fast and slow conditions, resulting in a total of 12 trials, consistent with Study 1.

Second, participants completed a forward digit span task adapted from Roembke and McMurray (2021). During this task, participants completed 4 trials in which they were required to recall sequences of 8 digits in order of appearance. In each trial, a participant viewed a sequence of 8 digits presented in the center of their screen. Participants were asked to mentally memorize the digits in order of appearance. Each digit was displayed for 1 s and disappeared before the next digit appeared. Immediately after a full sequence of eight digits was presented, participants were asked to type in the digits in order of appearance using their keyboard. Afterwards, participants were asked whether they wrote down the digits or used any external aids during the task.

Third, participants completed a verb–action matching task with iconic gesture cues (without iconic speech cues). This task used six unique verb–action pairs, which were different from those that appeared in the verb–action matching task with iconic speech cues, with each verb–action pairing appearing in both fast and slow conditions, resulting in a total of 12 trials, consistent with Study 1.

Three attention checks were administered throughout the testing session: one before, one between, and one after the verb–action matching tasks. In each attention check, participants listened to an audio clip and typed in the number of beeps they heard in this clip. Finally, participants answered demographic questions about their age, gender, ethnicity, and language background.

#### Counterbalancing and randomization

The counterbalancing of target action position, target speed (fast or slow), and whether the fast or slow version served as the target was similar to Study 1, with adjustments to exclude the normal-speed filler trials. Specifically, half of the target actions were manipulated with a fast speed and half with a slow speed, rather than the one-third distribution used in Study 1.

The verb–action pairings were presented in a prerandomized, fixed order for all participants. Within each task, the target speed (fast or slow) for a specific action-verb pairing was counterbalanced across two blocks. This resulted in two versions of the task. Participants were randomly assigned to one version of the task, with an equal number of participants completing each version.

#### Data analysis

The data analysis approach for Study 2 was the same as for Study 1, except for the additional forward digit span task. For the forward digit span task, we measured the proportion of correct digits in relation to the sequence of digits presented in the task. A digit entry was considered correct when both the digit itself and its position matched with the original sequence, following Roembke and McMurray (2021). Participants received a score of 1 for a correct digit, and a score of 0 for an incorrect digit. These scores were then summed across all digits of the same sequence and divided by the total number of digits in this sequence (*n* = 8) to create a proportion of correct digit entries for a given sequence. For instance, if a participant viewed a sequence of 8 digits as “41236785” and entered “41236785,” the proportion of the correct digits was .625. This calculation considered only the first four digits “4123” and the last digit “5” as correct (i.e., both the digit and position had to match the original sequence of 8 digits). Finally, the proportions of correct digits across the forward digit span trials were summed and divided by the total number of trials (*n* = 4) to create an average proportion of correct digits.

To test H1a, H1b, and H2a, we used the exact same analyses as in Study 1. To test H2b, we conducted a partial Spearman’s rank correlation analysis to examine the relationship between the average proportions of target action choices in the two verb–action matching tasks, while controlling the proportion of correct digits in the forward digit span task. Additionally, the following R packages were used: *ppcor* (Kim, 2015) for partial Spearman’s rank correlation analysis and *boot* (Canty & Ripley, 2022; Davidson & Hinkley, 1997) for the bootstrapped 95% confidence intervals.

### Results

#### Chance comparisons

**Fig. 4:**
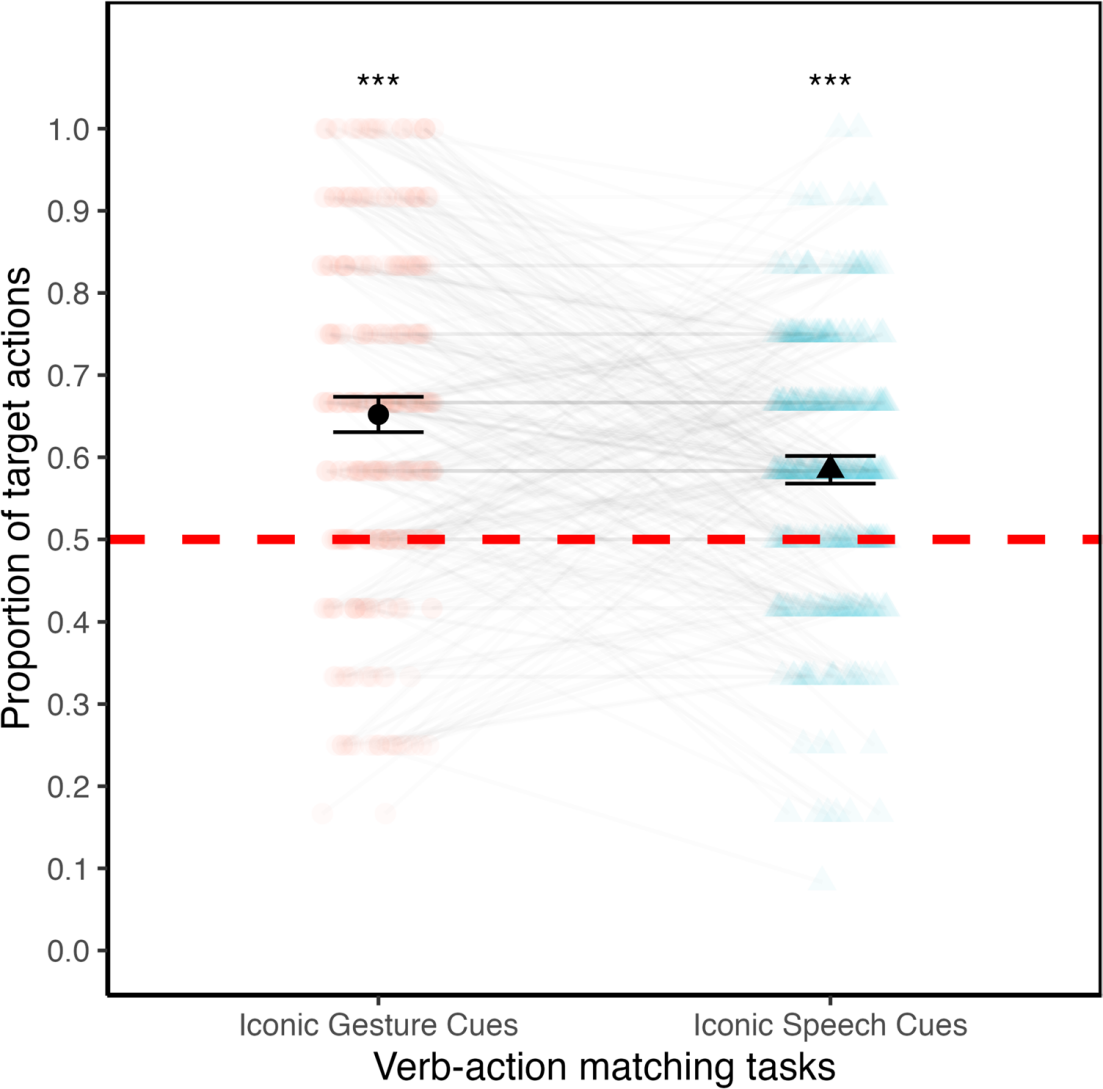
Average proportion target action choices by verb–action matching tasks (*N* = 348). *Notes.* Average performances (in proportion on the *y*-axis) organized by verb–action matching tasks (*x*-axis). The black shapes represent the means of all participants. Faded shapes represent individual performances, with light gray lines connecting the performances of the same individuals across the two verb–action matching tasks. Error bars represent 95% confidence intervals around the means. The dashed red horizontal line represents the chance level. *** represent significance level for each comparison of the mean performance in each verb–action matching task with chance (*p* < .001). *N* = 348 adult participants. (Color figure online)

To test H1a and H1b, we first compared adults’ average performances against chance (test value = .50) for each verb–action matching task. Figure 4 shows participants’ performances (in proportion) organized by verb–action matching tasks. The average proportion of target action choices of .58 (*SD* = .16) in the verb–action matching task with iconic speech cues was significantly above chance, *t*(347) = 9.89, *p* < .001, 95% CI around the mean [.56, .60]. The magnitude of this effect was moderate, Cohen’s *d* = 0.53, 95% CI *d* [0.41, 0.65]. The average proportion of target action choices of .65 (*SD* = .20) in the verb–action matching task with iconic gesture cues was also significantly above chance, *t*(347) = 13.85, *p* < .001, 95% CI around the mean [.63, .67]*.* The magnitude of this effect was moderate, Cohen’s* d* = 0.74, 95% CI *d* [0.63, 0.87].

#### Correlations and partial correlation

**Fig. 5:**
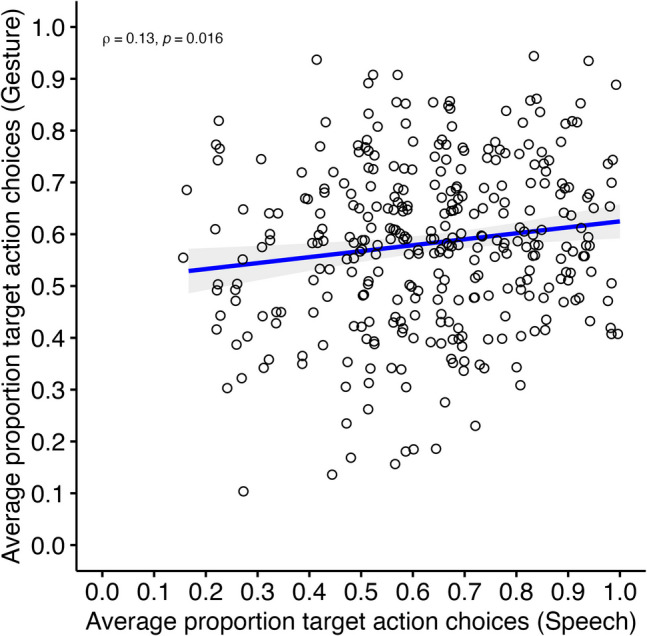
Spearman rank correlation between adults’ performances in the two verb–action matching tasks (Study 2, *N* = 348). *Note.* Scatterplot of the average proportion target action choices in the verb–action matching tasks with iconic gesture cues (*y*-axis) and iconic speech cues (*x*-axis). The jittered blank dots represent individual performances in both tasks. The thick blue line indicates the line of best fit, with the gray shaded area representing the 95% confidence intervals. Asterisk (*) represents the significance (*p* < .05) of Spearman’s rank correlation coefficient*. N* = 348 adult participants (Study 2). (Color figure online)

To test H2a, we correlated adults’ performances between the two verb–action matching tasks. Figure 5 illustrates the relationship between the average proportions of target action choices in the verb–action matching tasks with iconic speech cues and iconic gesture cues. There was a weak positive but significant correlation between verb–action matching tasks with iconic speech cues and iconic gesture cues, *ρ* (346) = .129, *p* = .016, 95% bootstrapped CI *ρ* [.021, .237].

To test H2b, we correlated adults’ performances between the two verb–action matching tasks, but this time we controlled verbal working memory. Table 1 shows the correlations between the three variables. Even when controlling the average proportion of correct digits in the forward digit span task, the weak positive but significant relationship between the performances in the verb–action matching tasks with iconic speech cues and iconic gesture cues persisted, *ρ *(346) = .136,* p* = .014, 95% bootstrapped CI *ρ* [.033, .240].

**Table 1:** Spearman rank correlations between the three task performances (*N* = 348)

Task performance	1	2	3
1. Verb–action matching (Speech)	–	.129* [.021, .237]	.124* [.024, .227]
2. Verb–action matching (Gesture)		–	− .018 [− .121, .082]
3. Forward digit span			–

*p < .05. 95% bootstrapped CIs around *ρ* are reported in square brackets. *N* = 348 adult participants

Finally, exploratory analyses of participants’ reaction times (preregistered) did not reveal any speed–accuracy trade-off effects (see Supplementary Materials).

### Discussion

Study 2 yielded two main findings. First, as in Study 1, participants successfully interpreted both iconic speech and gesture cues, selecting the target actions significantly above chance in both tasks, replicating the findings and confirming H1a and H1b. Second, Study 2 replicated the weak positive correlation between performances on the verb–action matching tasks with iconic speech and gesture cues, confirming H2a. This correlation remained significant even after controlling participants’ verbal working memory performance. This supports H2b and indicates that there is a specific cognitive mechanism for interpreting iconicity, rather than a general cognitive ability. Additionally, a weak but significant positive correlation between performances on the verb–action matching task with iconic speech cues and the verbal working memory task suggests that linguistic processing of iconic speech cues may engage verbal working memory. The overall performance in Study 2 was lower than in Study 1, possibly due to differences in environment (in-person vs. online) and participant populations (Prolific participants vs. students).

## General discussion

There are three key findings. First, adults inferred novel verb meanings from iconic speech or gesture cues conveying speed information (i.e., fast or slow speech or gesture rates), selecting the target actions significantly above chance. Thus, adults can derive verb meaning through iconicity, particularly regarding speed, from both speech (cross-modal mapping) and gesture (unimodal mapping). Second, adults’ performances in the two verb–action matching tasks, with either iconic speech or gesture cues, showed a weak positive correlation. This supports the hypothesis of a modality-independent mechanism for interpreting both unimodal and cross-modal iconicity. Third, this relationship persisted after controlling verbal working memory. This indicates that the effect is due to a specific cognitive mechanism for interpreting iconicity, rather than reflecting general cognitive abilities or levels of engagement with an online experiment. Together, these findings provide evidence for a modality-independent cognitive mechanism for detecting form-meaning resemblances.

The findings support a two-stage model of iconicity mapping: First, modality-specific processes convert sensory information (e.g., speed in the visual field) into schematic representations (e.g., temporal patterns such as rates of change), which is a process that happens separately for each modality. In a second stage, these representations are then compared across modalities to assess similarity and establish form-meaning correspondences. This mechanism may extend to other perceptual dimensions, such as size and intensity. For example, size can be iconically represented by oral cavity size during articulation (e.g., Sapir, 1929) and by gestural movement size (Beattie & Shovelton, 2002; Holler et al., 2009). Similarly, the “bouba–kiki” effect (Köhler, 1947) applies not only to shapes, but also to changes of color and emotion (Kantartzis, 2011). Thus, iconicity detection may depend on abstract representations that transcend our sensory experiences, which may partly explain why infants and great apes are less proficient than children and adults (Aussems & Moore, in press; Aussems et al., 2024; Bohn et al., 2019; Fort et al., 2018; Namy et al., 2000).

Our findings may challenge perspectives that view sound symbolism as similar to synesthesia or simple cross-sensory association (e.g., Ramachandran & Hubbard, 2001). Synesthesia and cross-sensory association are often considered low-level processes, where sensory experiences are automatically and involuntarily linked. In contrast, our results suggest that iconicity interpretation involves a higher-level cognitive process that deliberately integrates form-meaning mappings across multiple sensory inputs, based on abstract modality-independent representations.

The ability to interpret iconic speed information from speech rate when interpreting novel verb meanings is consistent with previous findings on prosodic cues. For example, English-speaking adults identified Japanese antonym pairs more accurately when spoken with expressive rather than monotone voice (Kunihira, 1971). Similarly, adults integrated prosodic speed cues, specifically speech rates, and recognized corresponding motion or static objects more quickly (Shintel & Nusbaum, 2007). Additionally, children as young as 4 years old interpreted iconic speed information conveyed by speech rate, highlighting the developmental roots of this interpretive ability (Hupp & Jungers, 2013).

Furthermore, our finding that adults can interpret iconic speed information conveyed by gesture rate when interpreting the meaning of a novel verb is also consistent with previous gesture comprehension studies. Adults glean additional information from co-speech iconic gestures, which are typically semantically related to the accompanying speech and depict a concrete referent (for a meta-analysis, see Dargue et al., 2019; Hostetter, 2011).

Our findings go beyond previous research on the role of iconic gestures in verb comprehension and generalization. Previous research mainly focused on how adults benefited from observing iconic gestures that depicted manner or path of locomotion when learning new words (e.g., Macedonia et al., 2011). To our knowledge, this study is the first to investigate whether adults interpret iconic gestures representing speed conveyed through gesture rates, and whether they use this information when comprehending the meaning of a novel verb. Additionally, many verb learning studies with children have focused on iconic gestures depicting manners of movement (e.g., Aussems & Kita, 2021; Aussems et al., 2022; Goodrich & Hudson-Kam, 2009; Mumford & Kita, 2014; Wakefield et al., 2018) and the end state of a change (Mumford & Kita, 2014), but not speed of movement, often conflating speed with other aspects of action, so our findings add promise for future developmental research in this area with children. Moreover, while verbal working memory was controlled in the current study, future research could explore whether other types of working memory, such as visuospatial (Özer & Göksun, 2020; Özer et al., 2023), or kinematic (Wu & Coulson, 2015), interact differently with how adults process iconic mappings across modalities.

## Supplementary Information

Below is the link to the electronic supplementary material.

Supplementary file1 (DOCX 3.23 MB)

## Data Availability

The raw data and study materials are available via the Open Science Framework (https://osf.io/4mnqg/).

## Code availability

The data analysis scripts in R are available via the Open Science Framework (https://osf.io/4mnqg/).

## Declarations

### Open practices statement

The data, materials, and analysis scripts for both studies are available (https://osf.io/4mnqg/), and Study 2 was preregistered (https://osf.io/jp6qn/).
