# Supplementary material for: Adults interpret iconicity in speech and gesture via the same modality-independent process
Source: Psychon Bull Rev. 2025 May 12;32(5):2331–43. doi: 10.3758/s13423-025-02698-2 (PMC12426134; doi:10.3758/s13423-025-02698-2)
Supplement: Supplementary file 1 — Supplementary file1 (DOCX 3.23 MB) [file 13423_2025_2698_MOESM1_ESM.docx]

**Supplementary Materials**

Three supplementary analyses were conducted to further explore the data: (a) pairwise comparisons of speed conditions, specifically fast vs. normal and normal vs. slow in Study 1 to validate our stimuli, (b) pairwise comparisons of the two verb-action matching task performances in Study 1 and Study 2, (c) an additional correlation analysis between participants’ reaction times and performances in both verb-action matching tasks in Study 2 (preregistered). These analyses were aimed at providing additional context for the main findings.

**Pairwise Comparisons of the Speed Conditions to Validate the Stimuli in Study 1**

To validate our stimuli in Study 1, we examined all speed conditions (Figure S1) and conducted two-tailed paired t-tests with Holm-Bonferroni correction for multiple comparisons, comparing fast vs. normal speed and slow vs. normal speed conditions for both verb-action matching tasks. Since the normal speed condition does not have a corresponding target action video, we instead calculated the proportion of fast action choices across all speed conditions. Specifically, participants received a score of 1 when choosing the fast action video and a score of 0 when choosing the slow action video. We then calculated the proportion of fast action choices by summing the scores and dividing them by the number of valid trials for each participant. These analyses confirmed that adults chose significantly more fast action videos in the fast speed condition (iconic speech cues: *M* = .76, *SD* = .28 ; iconic gesture cues: *M* = .84, *SD* = .20) than in the normal speed condition (iconic speech cues: *M* = .61, *SD* = .29 ; iconic gesture cues: *M* = .56, *SD* = .26), *t* (39) = 2.42, *p* = .020, 95% CI around the mean difference [.02, .28], and *t* (39) = 5.82, *p* < .001 , 95% CI around the mean difference [.18, .38], respectively. Adults also chose more fast action videos in the normal speed condition than in the slow speed condition (iconic speech cues: *M* = .14, *SD* = .22; iconic gesture cues: *M* = .19, *SD* = .26), *t* (39) = 8.02, *p* < .001, 95% CI around the mean difference [.35, .58], and *t* (39) = 6.07, *p* < .001, 95% CI around the mean difference [.25, .49]. Thus, this analysis validates our speed conditions for both the iconic speech and gesture stimuli.

**Figure S1**

*Average Proportion Fast Action Choices Across Speed Conditions by Verb-Action Matching Tasks (Study 1, N = 40)*


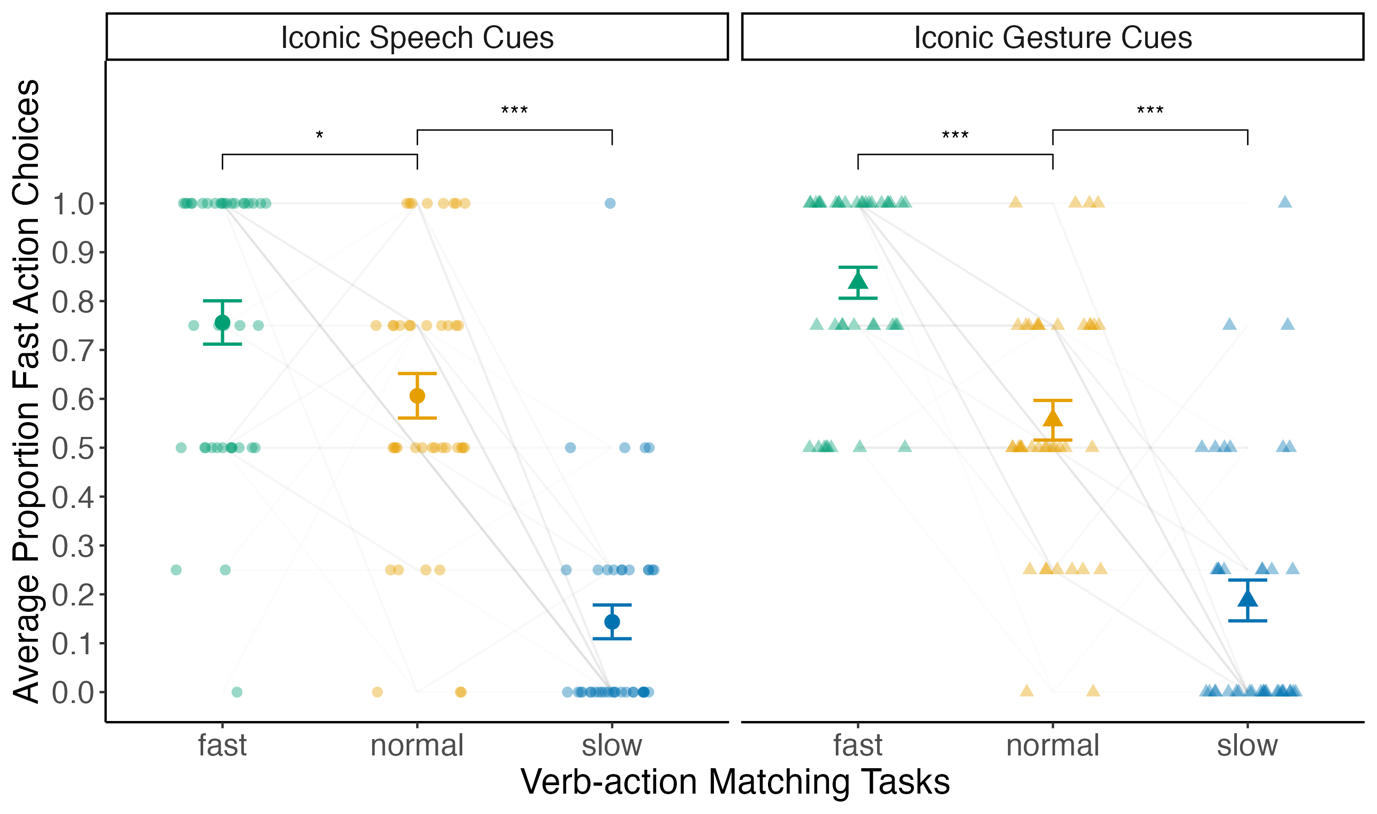
*Note*. Average proportion of fast action choices (in proportion on the *y*-axis) organized by iconic speed conditions (*x*-axis). The left panel represents performance in the verb-action matching task with iconic speech cues, and the right panel represents performance in the verb-action matching task with iconic gesture cues. Large shapes (circles, triangles) represent the means across all participants. Faded shapes (circles, triangles) represent individual performances, with light gray lines connecting the performances of the same individuals across the three iconic speed conditions within the same verb-action matching task. Error bars represent 95% confidence intervals around the means. Asterisks represent the significance for pairwise t-test comparisons of the mean performances between fast vs. normal and normal vs. slow, *p* < .05 (*), *p* < .001 (***). *N* = 40 adult participants (Study 1).

**Pairwise Comparisons Between the Verb-Action Matching Task Performances**

Next, we compared performances between the two verb-action matching tasks in both Study 1 and 2, to explore potential differences in adults’ understanding of iconic mappings in speech and gesture. We conducted two paired t-tests (two-tailed) with Holm-Bonferroni correction for multiple comparisons. The overall performance was lower in the verb-action matching task with iconic speech cues compared to verb-action matching task with iconic gesture cues in both Study 1 and 2. However, this difference was not statistically significant in Study 1, *t* (39) = 0.78, *p* = .440, 95% CI around the mean difference [-.09, .04], whereas it was statistically significant in Study 2, *t* (347) = 5.22, *p* < .001, 95% CI around the mean difference [.04, .09].

One possible interpretation for why participants showed higher performance with iconic gesture cues than iconic speech cues in Study 2 is that participants were exposed longer to the iconic gesture cues (which were played in videos on loop) compared to the iconic speech cues, giving them more time to detect the iconic relationship between the cue and actions in the videos. However, this finding should be interpreted with caution, as our study was not designed for direct comparison between the two task performances, because the task order was not counterbalanced. Without counterbalancing, it is not possible to distinguish whether differences in performance reflect modality-specific effects in detecting iconicity in speech or gesture or the influence of task order.

**Correlation between Reaction Times and Verb-Action Matching Task Performances**

Finally, we examined whether adults’ performances in the two verb-action matching tasks showed any speed-accuracy trade-off effects in Study 2 (pre-registered). There were no significant correlations between participants’ reaction times and their performance in the verb-action matching task with iconic speech cues, *ρ* (346) = −.01, *p* = .065, bootstrapped 95% CI *ρ* [−.21, .00], or with iconic gesture cues, *ρ* (346) = .01, *p =* .876, bootstrapped 95% CI *ρ* [−.09, .12]. Thus, no significant speed-accuracy trade-off effects were observed in either of the verb-action matching tasks.
